# Circular RNAs in Intervertebral Disc Degeneration: An Updated Review

**DOI:** 10.3389/fmolb.2021.781424

**Published:** 2022-01-06

**Authors:** Derong Xu, Xuexiao Ma, Chong Sun, Jialuo Han, Chuanli Zhou, Sunny Hei Wong, Matthew T. V. Chan, William K. K. Wu

**Affiliations:** ^1^ Department of Spine Surgery, The Affiliated Hospital of Qingdao University, Qingdao, China; ^2^ Lee Kong Chian School of Medicine, Nanyang Technological University, Singapore, Singapore; ^3^ Department of Anaesthesia and Intensive Care and Peter Hung Pain Research Institute, The Chinese University of Hong Kong, Hong Kong, China; ^4^ State Key Laboratory of Digestive Diseases, LKS Institute of Health Sciences, The Chinese University of Hong Kong, Hong Kong, China

**Keywords:** CircRNAs, lower back pain, ncRNAs, intervertebral disc degeneration, CircITCH

## Abstract

Low back pain, a common medical condition, could result in severe disability and inflict huge economical and public health burden. Its pathogenesis is attributed to multiple etiological factors, including intervertebral disc degeneration (IDD). Emerging evidence suggests that circular RNAs (circRNAs), a major type of regulatory non-coding RNA, play critical roles in cellular processes that are pertinent to IDD development, including nucleus pulposus cell proliferation and apoptosis as well as extracellular matrix deposition. Increasing number of translational studies also indicated that circRNAs could serve as novel biomarkers for the diagnosis of IDD and/or predicting its clinical outcomes. Our review aims to discuss the recent progress in the functions and mechanisms of newly discovered IDD-related circRNAs.

## 1 Introduction

Low back pain (LBP) is one of the most common medical conditions. It can cause severe incapacity at the individual level and impact the workforce and the health care system at the societal level (Abbasi et al., 2021; Aransay et al., 2020; Martín-Corrales et al., 2020). Much effort has been taken to study the mechanism underlying the pathogenesis of LBP and to improve its treatment (Shi et al., 2020; Mera et al., 2021; Umimura et al., 2021). The etiology of LBP involves multiple factors, among which intervertebral disc degeneration (IDD) is a widely known cause (Abdollah et al., 2020; Ishitani et al., 2020; Shi et al., 2020; Huang et al., 2021a; Zhang et al., 2021a; Liu et al., 2021). IDD is a chronic process resulting in structural failure of the intervertebral disc, with accelerated or advanced signs of aging. Etiologically, IDD may be hastened by co-morbidities (e.g., diabetes and obesity), certain lifestyles (e.g., smoking, occupation, alcohol consumption), aging, and genetic predisposition (Tang et al., 2020; Tsingas et al., 2020; Yang et al., 2021a; Baldia et al., 2021; Shao et al., 2021; Zhao et al., 2021). The mainstay of treatment for IDD-related diseases is surgical intervention. Nucleus pulposus (NP), which is a core structural component of the intervertebral disc, is consisted of extracellular matrix (ECM) and NP cells (Jiang et al., 2019a; Bach et al., 2019; Kang et al., 2019; Ohnishi et al., 2019; Hanaei et al., 2020). Deregulated functions of NP cells, such as aberrant cell apoptosis, proliferation, and ECM degradation/synthesis, have been demonstrated to contribute to IDD development (Li et al., 2020a; Li et al., 2020b; Gao et al., 2020; He et al., 2020; Zhao et al., 2021).

Circular RNAs (circRNAs) are a major type of non-coding regulatory RNAs that are produced by non-canonical back-splicing events (Li et al., 2019a; Liu et al., 2020; Ma et al., 2020; Li et al., 2021a; Li et al., 2021b). CircRNAs are known to modulate gene expression principally via sponging microRNAs (miRNAs) (Li et al., 2020c; Pan et al., 2020; Ni et al., 2021). In addition, some circRNAs could mediate their biological functions through direct interactions with proteins or inhibiting mature mRNA formation (Tian et al., 2019; Tu et al., 2020; Li et al., 2021c). A minor subset of circRNAs also harbor coding potential for translation into protein through rolling circle amplification (Jiang et al., 2019b; Jin et al., 2019; Long et al., 2020). Growing evidence suggests that circRNAs play critical roles in different cellular processes including cell proliferation, apoptosis, differentiation, migration, and metabolism (Cai et al., 2018; Zhang et al., 2018; Liang et al., 2019; Yu et al., 2019; Guo et al., 2020a; Li et al., 2020d). A myriad of circRNAs have also been identified to be deregulated in human diseases, such as cancers, cardiovascular diseases, congenital diseases, and IDD (Chen et al., 2019; Pan et al., 2019; Li et al., 2020c). Translational studies also indicate that circRNAs could act as novel diagnostic and prognostic biomarkers for early disease detection and predicting the clinical outcomes of some diseases, including IDD (Liu et al., 2019; Li et al., 2020e; Yang et al., 2021b; Guo et al., 2021).

We previously reviewed the involvement of circRNAs in NP cell biology and IDD pathogenesis (Li et al., 2019b). Since then, more than a dozen of circRNAs had been identified to be associated with IDD. In the present work, we would like to summarize the functions and mechanisms of these newly discovered IDD-related circRNAs that have not been covered by our previous review. The potential prognostic and therapeutic utilities of these newly identified circRNAs in IDD will also been discussed.

## 2 Common Approaches for the Identification and Functional Characterization of IDD-Related circRNAs

Transcriptome sequencing is the most widely used method for the identification of disease-related circRNAs. Nevertheless, due to the overlap with sequence of the linear RNA transcribed from the same gene, additional processing workflows (e.g., linear RNA removal through exonuclease digestion) and specific computational algorithms (e.g., CIRI2, DCC, Sailfish-cir, CIRIquant) are required to such back-spliced reads (Cheng et al., 2016; Li et al., 2017; Gao et al., 2018; Zhang et al., 2020). Microarrays with probes specifically targeting the back-splice sites have also been developed for circRNA profiling (Li et al., 2019c) (29415187). Following the initial discovery, the differential expression of circRNAs can be confirmed by reverse transcription-quantitative PCR (RT-qPCR) using divergent primers spanning the back-splice junction sequence (Panda and Gorospe, 2018). In IDD studies, most commonly used samples for differentially expressed circRNA discovery are degenerative discs from IDD patients with normal discs from cadaveric donors or those from patients suffering from vertebral fracture as controls. Some investigators also profiled circRNAs in the cartilage endplates. For functional characterization of the identified circRNAs, gain-of-function (i.e., overexpression) and loss-of-function (i.e., silencing with small interfering RNA) approaches have been done in cultured NP cells or chondrocytes with or without further validation in animal models of IDD.

## 3 Functions and Mechanisms of Action of Newly Discovered circRNAs in IDD

### 3.1 CircSNHG5

Like the NP cells, dysfunction of chondrocytes in the cartilage endplates has been implicated in IDD. Zhang et al. (2021b) studied the functional role of circSNHG5 in IDD-associated cartilage endplates. The authors showed that circSNHG5 was downregulated in the degenerative cartilage endplates as compared with healthy cartilage endplates. Functionally, knockdown of circSNHG5 inhibited chondrocyte proliferation and drove the degradation of collagen II and aggrecan. Moreover, they showed that circSNHG5 sponged miR-495-3p expression to derepress the downstream gene CITED2. Their data suggested that deregulation of the circSNHG5/miR-495-3p/CITED2 axis contributes to IDD development.

### 3.2 CircARL15

The loss of balance between proliferation and apoptosis of NP cells could contribute to NP cell loss during IDD. Wang et al. (2021) showed that circARL15 was one of the most downregulated circRNAs within the competing endogenous RNA (ceRNA) network in IDD. CircARL15 level was decreased whereas miR-431-5p level was increased in IDD samples, in which their expression showed significant negative correlation with each other. Ectopic expression of circARL15 enhanced NP cell proliferation and suppressed NP cell apoptosis. Mechanistically, circARL15 was shown to sponge miR-431-5p to disinhibit DISC1 to mediate its protective effects on NP cells. These data suggested that the aberrant downregulation of circARL15 contributes to IDD through promoting NP cell death via the miR-431-5p/DISC1 pathway.

### 3.3 CircITCH

Aberrant degradation of disc ECM components, such as aggrecan and type II collagen, is a hallmark of IDD. Zhang et al. (2021c) studied the role of circITCH in the degradation of ECM during IDD. They found that circITCH was overexpressed in the NP tissues from IDD patients compared to the control NP samples. Overexpression of circITCH suppressed NP cell proliferation and induced NP cell apoptosis. Enforced expression of circITCH also shifted the balance from ECM production to ECM degradation, characterized by decreased aggrecan and collagen II expression and increased MMP13 and ADAMTS4 expression. Mechanistically, circITCH sponged miR-17-5p to derepress SOX4 that promoted the Wnt/β-catenin signaling to accelerate ECM degradation in NP cells. Accordingly, miR-17-5p inhibitor, SOX4 overexpression and LiCl (a β-catenin/Wnt signaling activator) reversed ECM degradation and apoptosis induced by circITCH knockdown. These data suggested that circITCH induced ECM degradation through inducing the Wnt/β-catenin pathway via the miR-17-5p/SOX4 axis.

### 3.4 circPKNOX1

Similar to SOX4, SOX9 plays a key role in regulating NP cell function. Huang et al. (2021b) demonstrated that circPKNOX1 was downregulated in IDD cells as compared with control NP cells. Importantly, enforced expression of circPKNOX1 increased the levels of SOX9, aggrecan and collagen II and suppressed the expression of ECM-degrading enzymes, namely MMP13, MMP3, ADAMTS-5, and ADAMTS4. Furthermore, circPKNOX1 sponged miR-370-3p to restore the expression of KIAA0355. Their data suggested that reduced expression of circPKNOX1 promotes IDD development via promoting ECM degradation through the miR-370-3p/KIAA0355 axis. Thus, these data demonstrated that miR-370-3p may serve as a therapeutic target for IDD treatment.

### 3.5 Circ-FAM169A

The nuclear factor (NF)-κB-mediated signaling is an important pro-inflammatory pathway. Li et al. (2021d) showed that circ-FAM169A was overexpressed in degenerative NP samples compared to control NP tissues. Overexpression of circ-FAM169A significantly increased ECM degradation and inhibited ECM synthesis in NP cells. Moreover, they found that circ-FAM169A sequestered miR-583 to restore BTRC expression, which is an inducer of the NF-κB pathway. These data suggested that circ-FAM169A may induce IDD progression through miR-583/BTRC signaling. Concordantly, Li et al. (2021d) found that circ-FAM169A was overexpressed whereas miR-583 was downregulated in the degenerative NP tissues, in which their expression showed significant negative correlation with each other. They then reconstructed the circ-FAM169A/miR-583/mRNA ceRNA network and suggested that the circ-FAM169A/miR-583 pathway may play important roles in regulating apoptosis and ECM metabolism of NP cells. Furthermore, they showed that circ-FAM169A directly sponged miR-583 to derepress SOX9. These two studies suggested that the circ-FAM169A-miR-583 axis is involved in IDD pathogenesis.

### 3.6 CircGLCE

Chen et al. (2020) showed that circGLCE was localized in the cytoplasm of NP cells and it was decreased in the IDD tissues. Functionally, silencing of circGLCE was found to promote the expression of ECM-degrading enzymes and induce apoptosis of NP cells. Mechanistically, circGLCE sponged miR-587 expression in NP cells to derepress STAP1. The protective effect of circGLCE was also confirmed *in vivo*. Collectively, these data showed that circGLCE could suppress IDD development through inhibiting NP cell apoptosis and ECM degradation via targeting the miR-587/STAP1 axis.

### 3.7 Circ_0059955

Like circITCH, circ_0059955 is another circRNA transcribed from the host gene ITCH (Itchy E3 ubiquitin protein ligase). Kong et al. (2020) demonstrated that circ_0059955 was significantly downregulated in IDD samples. Knockdown of circ_0059955 suppressed ITCH expression and inhibited proliferation, induced cell cycle arrest and promoted apoptosis of NP cells. Furthermore, they demonstrated that enforced expression of circ_0059955 ameliorated IDD development in a rat model. These data suggested that circ_0059955 could protect against IDD.

### 3.8 CircRNA_0000253

Exosomes are a major means for intercellular transfer of non-coding RNAs. Song et al. (2020) identified circRNA_0000253 as one of the most upregulated circRNAs in the exosomes released from the degenerative NP cells. Functionally, circRNA_0000253 could promote IDD development via downregulating SIRT1 and sponging miRNA-141-5p *in vitro* and *in vivo*. Their data suggested that the aberrant upregulation of exosomal circRNA_0000253 may contribute to IDD.

### 3.9 CircVMA21

XIAP (X linked inhibitor-of-apoptosis protein) is a potent inhibitor of apoptosis by binding to caspases 3, 7, and 9. Cheng et al. (2018) found that XIAP was downregulated in the degenerative NP samples and inflammatory cytokine-induced NP cells, where XIAP downregulation was correlated with the deregulated balance between ECM synthesis and degradation as well as excessive apoptosis. miR-200c could undermine NP cell viability via suppressing XIAP expression. circVMA21 was identified to sponge miR-200c to promote NP cell function via derepressing XIAP. Moreover, they demonstrated that circVMA21 alleviated IDD in the rat model. Their data suggested that circVMA21 may counteract inflammatory cytokine-induced imbalance between the catabolism and anabolism of ECM and NP cell apoptosis via regulating the miR-200c-XIAP axis.

### 3.10 Circ-GRB10

ERBB2 is a member of the human epidermal growth factor receptor family that transduces mitogenic and pro-survival signal. Guo et al. (2018) showed that circ-GRB10 was decreased in NP samples from IDD patients as compared to normal NP samples. Enforced expression of circ-GRB10 suppressed NP cell apoptosis. Mechanistically, circ-GRB10 targeted miR-328-5p to derepress ERBB2 to positively regulate cell proliferation. Circ-GRB10 suppressed IDD development in the rat model. Moreover, the upstream mechanism underlying circ-GRB10 dysregulation was elucidated. Guo et al. (2020b) found that FUS could promote circ-GBR10 biosynthesis in the NP cells in which FUS expression was regulated by miR-141-3p. Downregulation of ERBB2 was also found to suppress Erk1/2 phosphorylation that regulated miR-141-3p expression in NP cells, highlighting FUS and miR-141-3p as important modulators of circ-GRB10 synthesis. Their data suggested that circ-GRB10 could protect against IDD development and the downstream miR-328-5p might serve as a potential therapeutic target for IDD.

### 3.11 Circ-TIMP2

The gene *TIMP2* (Tissue Inhibitor Of Metalloproteinases 2) encodes a protein that serves as a natural inhibitor of the matrix metalloproteinases. Guo et al. (2020c) showed that circ-TIMP2, also transcribed from *TIMP2*, was overexpressed in IDD samples as compared to normal NP samples. Upregulation of circ-TIMP2 inhibited ECM synthesis and induced ECM degradation. In contrast, miR-185-5p suppressed ECM degradation induced by interleukin (IL)-1β and tumor necrosis factor (TNF)-α. Bioinformatic analysis showed that MMP2 was a potential target of miR-185-5p. Consistently, MMP2 was upregulated after exposure to IL-1β and TNF-α in NP cells, which could be rescued by transfecting with miR-185-5p mimic. Importantly, they demonstrated that circ-TIMP2 sponged miR-185-5p to induce ECM degradation. These data suggested that circ-TIMP2 could promote ECM degradation through the miR-185-5p/MMP2 signaling in NP cells.

### 3.12 circ_001653

Cui and Zhang (2020) showed that circ_001653 was upregulated in the degenerative NP cells and samples compared to control groups and was closely related to IDD severity. Overexpression of circ_001653 promoted NP cell apoptosis and resulted in an imbalance between catabolic and anabolic factors of ECM whereas miR-486-3p enhanced NP cell viability by suppressing CEMIP. Circ_001653 was found to sponge miR-486-3p to induce CEMIP to contribute to NP cell death. In mice, knockdown of circ_001653 alleviated IDD development. Their data suggested that targeting circ_001653 may be a novel therapeutic strategy the delay IDD development.

### 3.13 Circ-CIDN

Excessive mechanical loading can be transduced by NP cells. Xiang et al. (2020) showed that circ-CIDN was decreased in the compressed NP cells compared to control NP cells. Functionally, enforced expression of circRNA-CIDN rescued compression-induced NP cell apoptosis and ECM degradation. CircRNA-CIDN was found to sponge miR-34a-5p that could promote compression-induced damage via inhibiting SIRT1 expression. The protective effect of circ-CIDN against IDD was also confirmed in the IDD model. Their data suggested that circ-CIDN played an important role in mitigating mechanical loading-induced NP cell damage through regulating the miR-34a-5p/SIRT1 axis.

### 3.14 CircERCC2

Removal of damaged mitochondria through the autophagy (i.e., mitophagy) is key to cell survival. Xie et al. (2019) demonstrated that circERCC2 was one of the most downregulated circRNAs in IDD. Knockdown of circERCC2 promoted miR-182-5p expression and suppressed SIRT1 expression in the degenerative NP samples and tert-Butyl hydroperoxide (TBHP; oxidative stress inducer)-exposed NP cells, in which knockdown of SIRT1 inhibited mitophagy and promoted apoptosis. Moreover, miR-182-5p was found to regulate the mitophagy and NP cell apoptosis by targeting SIRT1. The protective effects of circERCC2 on NP cells and IDD rat model were mediated by the miR-182-5p/SIRT1 axis. This study indicated circERCC2 could ameliorate IDD via sponging miR-182 to derepress SIRT1 for activating mitophagy and inhibiting NP cell apoptosis. Restoring circERCC2 expression might be a potential therapeutic approach for IDD.

### 3.15 CircSEMA4B

Aberrant Wnt signaling is linked to IDD. Wang et al. (2018a) showed that circSEMA4B was decreased in the IDD samples compared to control tissues. Overexpression of circSEMA4B attenuated the effect of IL-1β on NP cell senescence, proliferation, and aggrecan degradation through the Wnt signaling in IDD. CircSEMA4B was found to sponge miR-431 to disinhibit GSK-3β and SFRP1. The effect of circSEMA4B inhibition on NP cells was partially rescued by the inhibition of miR-431. These findings suggested that circSEMA4B could suppressing IL-1β-induced degeneration via Wnt signaling in NP cells.

### 3.16 CircRNA_104670

Increased MMP2 activity has been reported in IDD (Rutges et al., 2008). Song et al. (2018) demonstrated that circRNA_104670 was upregulated in IDD NP tissues compared to normal tissues. circRNA_104670 and its downstream target could distinguish IDD from healthy NP with an area under the receiver-operating characteristic curve of 0.96 and 0.91, respectively. In additional, there are significant positive and negative correlation of Pfirrmann disc grade with the expression of circRNA_104670 and miR-17-3p, respectively. Functionally, knockdown of circRNA_104670 inhibited NP cell apoptosis and reduced MMP2 expression accompanied by enhanced ECM formation. CircRNA_104670 inhibition in mice also resulted in lower IDD grade, whereas miRNA-17-3p inhibition or circRNA_104670 overexpression in mice led to higher IDD grade. These data suggested that circRNA_104670 could serve as a prognostic marker and a therapeutic target in IDD.

### 3.17 Circ-4099

Aside from functioning as pathogenic mediators, differential expression of circRNAs could serve as an autoprotective mechanism. Wang et al. (2018b) demonstrated that circ-4099 was upregulated in the degenerated NP samples. Interestingly, enforced expression of circ-4099 induced aggrecan and collagen II expression and suppressed the release of pro-inflammatory factors, including prostaglandin E_2_, TNF-α and IL-1β. Upstream, TNF-α increased the level of circ-4099 in NP cells via upregulating GRP78, in which MAPK/NF-κB shRNAs or inhibitors abolished the induction of circ-4099 by TNF-α. Downstream, circ-4099 sponged miR-616-5p to derepress SOX9. These results suggested that circ-4099 acted as an autoprotective circRNA in IDD development.

### 3.18 circ_0075062

Insufficient supply of nutrition to NP cells has been implicated in IDD pathogenesis (De Geer, 2018). Chang et al. (2021) showed that circ_0075062 was overexpressed in the IDD NP samples and glucose-deprived NP cells. Using RNase tolerance assay coupled with sequencing, they found that circ_0075062 was confirmed to be a circular transcript. Knockdown of circ_0075062 alleviated ECM degradation in glucose-deprived NP cells. These results suggested that circ_0075062 may be a target for IDD.

## 4 Conclusion

**FIGURE 1:**
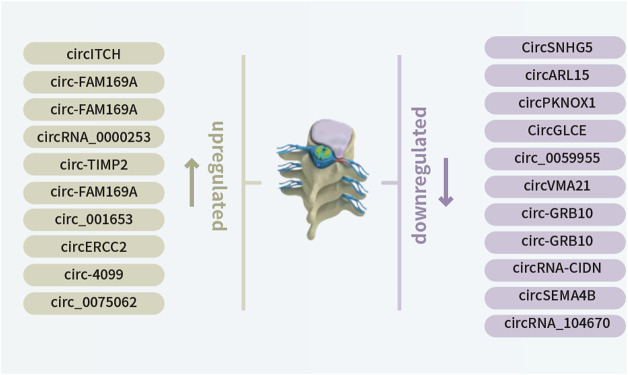
Newly identified differentially expressed circRNAs in IDD, including 10 downregulated and 11 upregulated circRNAs.

**FIGURE 2:**
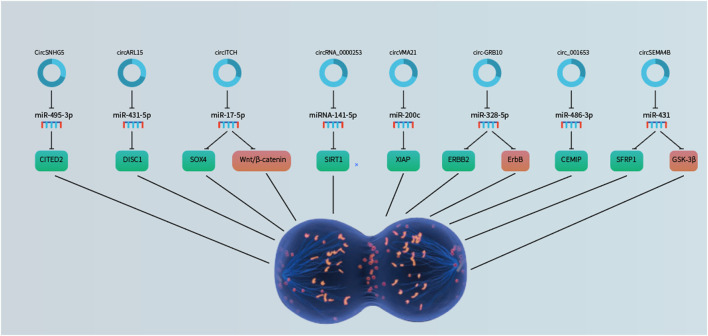
CircRNAs as regulators of NP cell proliferation through sponging microRNAs to derepress the downstream target genes.

**FIGURE 3:**
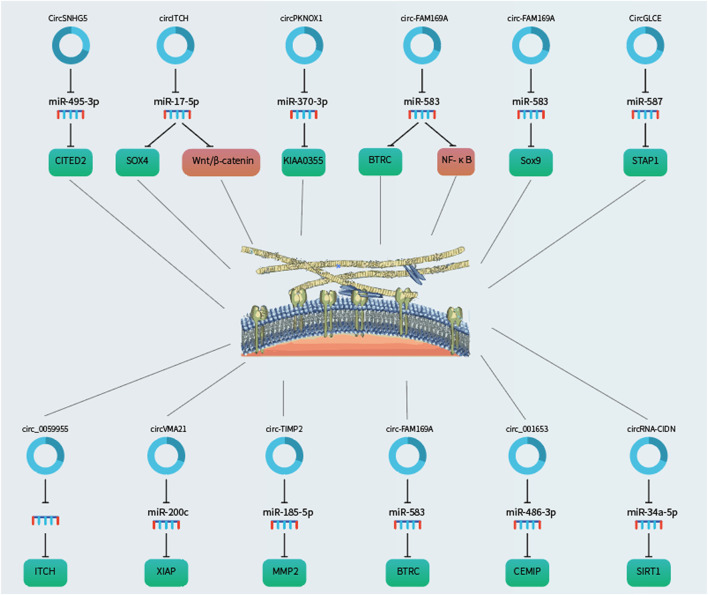
CircRNAs as regulators of ECM remodelling in the NP cells via sponging microRNAs to derepress the downstream target genes.

**FIGURE 4:**
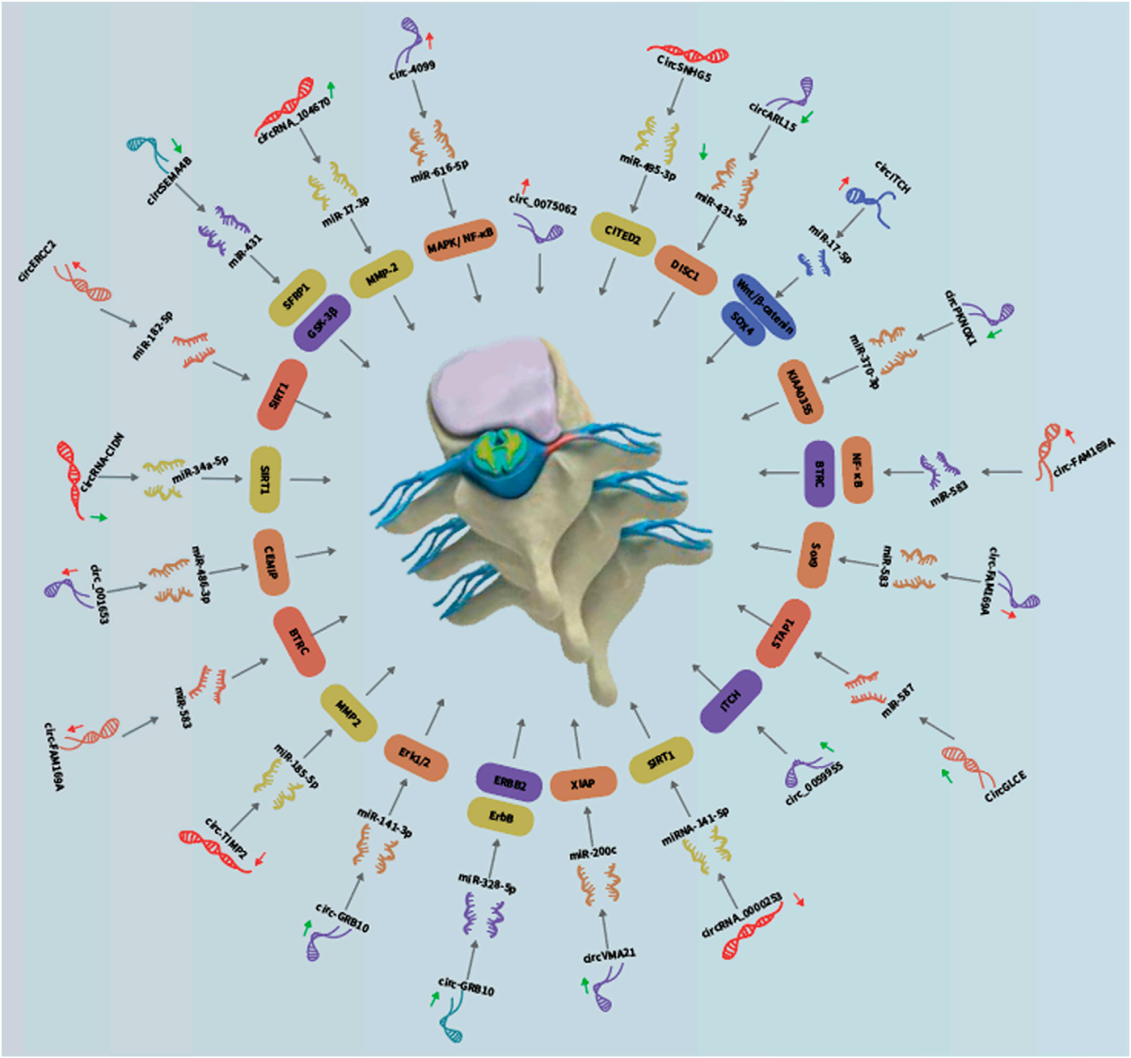
Crucial roles of circRNAs in IDD development.

IDD is contributed by deregulated NP cell functions, including aberrant apoptosis, proliferation, and ECM degradation/synthesis. Emerging studies have pointed to the involvement of non-coding RNAs, including miRNAs, long non-coding RNAs and circRNAs (Figures 1, 2, 3 and 4; Table 1), in IDD through their extensive crosstalk with NP cell function-related signaling pathways, such as Wnt and NF-κB signaling. Transcription regulators/factors, such as CITED2, SIRT1, and SOX4/9, also appeared to be heavily deregulated by circRNA-miRNA networks in IDD. To date, the upstream mechanism mediating the deregulation of most circRNAs in NP cells and their relationship with IDD risk factors, including mechanical loading, aging, smoking and genetic predisposition, remain largely unknown. Moreover, reports on deregulated circRNAs in other cell types pertinent to IDD, such as cartilage endplate chondrocytes, are scarce. For clinical translation, although some tissue circRNAs were shown to be able to discriminate IDD samples from healthy tissues, the use of circulating circRNAs as diagnostic markers have not been reported. Future research efforts should therefore be put forward to large-scale screening and validation of diagnosis/prognosis-related circulating IDD-related circRNAs. As therapeutic targets, upregulated circRNAs of pathogenic significance might be inhibited by RNA interference or antisense oligos. Disinhibited miRNAs downstream to downregulated circRNAs might also be targeted to achieve therapeutic effect. Nevertheless, tissue- or cell type-specific delivery of RNA-targeting agents remains technically challenging. In this connection, NP cell-targeting approaches, for instance with nanoparticles, are urgently needed. With these, it is hopeful that novel circRNA-based therapeutics and biomarkers will emerge to improve the clinical management of IDD.

**TABLE 1:** Dysregulated circRNAs in intervertebral disc degeneration

Name	Dysregulation	Sponge target	Function	Related gene	Role	Reference
CircSNHG5	Downregulated	miR-495-3p	Proliferation	CITED2	Protective	Zhang et al. (2021b)
			ECM			
circARL15	Downregulated	miR-431-5p	Proliferation apoptosis	DISC1	Protective	Wang et al. (2021)
circITCH	Upregulated	miR-17-5p	Apoptosis	SOX4	Harmfulness	Zhang et al. (2021c)
			ECM	Wnt/β-catenin		
			Proliferation			
circPKNOX1	Downregulated	miR-370-3p	ECM	KIAA0355	Protective	Huang et al. (2021b)
circ-FAM169A	Upregulated	miR-583	ECM	BTRC	Harmfulness	Li et al. (2021d)
				NF-κB		
CircGLCE	Downregulated	miR-587	ECM apoptosis	STAP1	Protective	Chen et al. (2020)
circ_0059955	Downregulated		Cycle apoptosis	ITCH	Protective	Kong et al. (2020)
			ECM			
circRNA_0000253	Upregulated	miRNA-141-5p	Proliferation	SIRT1	Harmfulness	Song et al. (2020)
circVMA21	Downregulated	miR-200c	Proliferation	XIAP	Protective	Cheng et al. (2018)
			Apoptosis			
			ECM			
circ-GRB10	Downregulated	miR-328-5p	Apoptosis	ERBB2	Protective	Guo et al. (2018)
			Proliferation	ErbB		
circ-GRB10	Downregulated	miR-141-3p	Apoptosis	Erk1/2	Protective	Guo et al. (2020b)
circ-TIMP2	Upregulated	miR-185-5p	ECM	MMP2	Harmfulness	Guo et al. (2020c)
circ-FAM169A	Upregulated	miR-583	ECM	BTRC	Harmfulness	Guo et al. (2020d)
circ_001653	Upregulated	miR-486-3p	ECM	CEMIP	Harmfulness	Cui and Zhang (2020)
			Proliferation			
			Migration apoptosis			
circRNA-CIDN	Downregulated	miR-34a-5p	Apoptosis ECM	SIRT1	Protective	Xiang et al. (2020)
circERCC2	Upregulated	miR-182-5p	Apoptosis mitophagy	SIRT1	Harmfulness	Xie et al. (2019)
circSEMA4B	Downregulated	miR-431	Senescence proliferation	SFRP1	Protective	Wang et al. (2018a)
			ECM	GSK-3β		
				Wnt		
circRNA_104670	Downregulated	miR-17-3p	ECM	MMP-2	Protective	Rutges et al. (2008)
circ-4099	Upregulated	miR-616-5p	Inflammatory factors secretion	MAPK/NF-κB	Harmfulness	Song et al. (2018)
			ECM			
circ_0075062	Upregulated		ECM		Harmfulness	Wang et al. (2018b)
